# Supplementary material for: Construction of CNT-MgO-Ag-BaO Nanocomposite with Enhanced Field Emission and Hydrogen Sensing Performances
Source: Nanomaterials (Basel). 2023 Feb 27;13(5):885. doi: 10.3390/nano13050885 (PMC10005578; doi:10.3390/nano13050885)
Supplement: Supplementary file 1 [file nanomaterials-13-00885-s001.zip › nanomaterials-2192903-supplementary.pdf]

Supplementary Material

Construction of CNT–MgO–Ag–BaO Nanocomposite with Enhanced Field Emission and Hydrogen Sensing Performances

Xingzhen Liu, Weijin Qian *, Yawei Chen, Mingliang Dong, Taxue Yu, Weijun Huang and Changkun Dong *

Wenzhou Key Lab of Micro-Nano Optoelectronic Devices, Wenzhou University, Wenzhou 325035, China; lxz092799@163.com (X.L.); w2549117790@163.com (Y.C.); dml13946319580@126.com (M.D.); 13646557519@163.com (T.Y.); 18857757816@163.com (W.H.)

* Correspondence: weijinqian@wzu.edu.cn (W.Q.), dck@wzu.edu.cn (C.D.); Tel.: +86-577-86689067 (C.D.)

Index

SI-1. J-E curves of the CNT-based samples with three times FE tests.

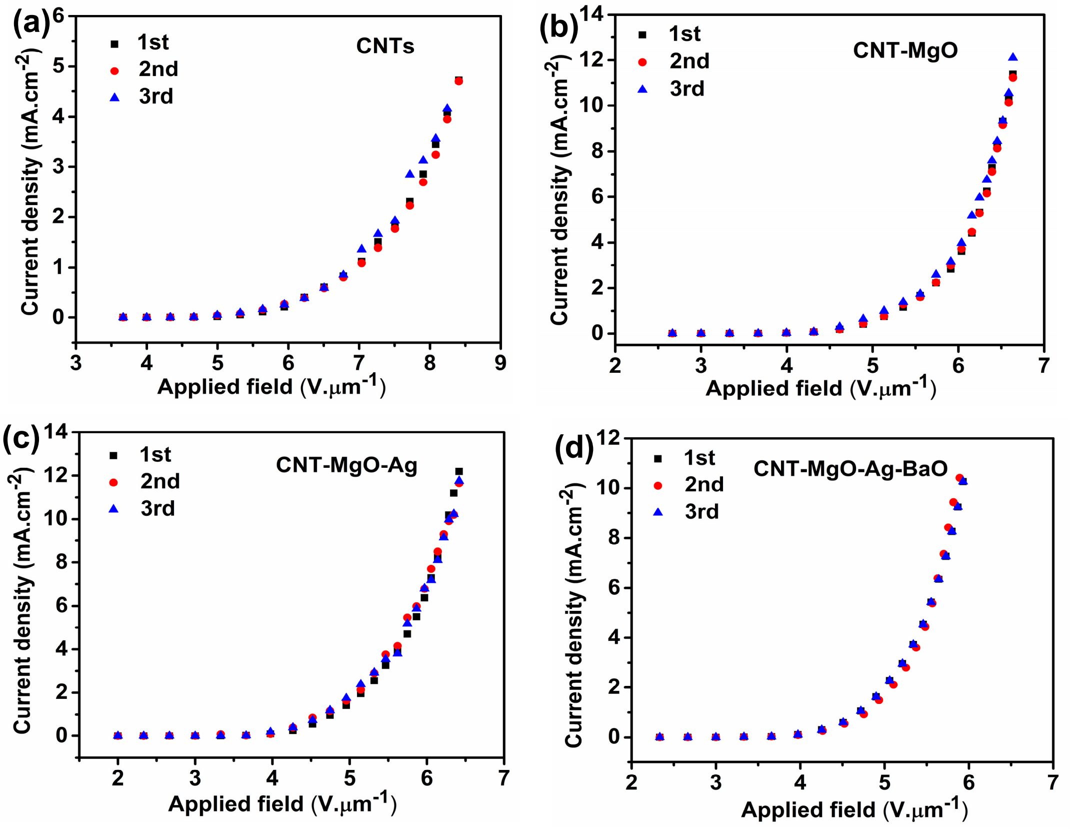

Figure S1. J-E curves of the CNT-based samples with three times FE tests. (**a**) the pristine CNTs; (**b**) CNT-MgO composite; (**c**) CNT-MgO-Ag composite; (**d**) CNT-MgO-Ag-BaO composite.
